# Supplementary material for: WordCommentsAnalyzer: A windows software tool for qualitative research
Source: F1000Res. 2018 Sep 4;7:536. Originally published 2018 May 3. [Version 2] doi: 10.12688/f1000research.14819.2 (PMC6137411; doi:10.12688/f1000research.14819.2)
Supplement: Supplementary file 3 [file f1000research-7-17614-s0002.tgz › b2a79496-4ada-47c4-9682-a99ce867836d.zip!/Eight Iranian Health Journals/Archives of Rehabilitation_60.docx]

© Archives of Rehabilitation. This work is licensed under CC BY-NC 4.0 (https://creativecommons.org/licenses/by-nc/4.0/).

Volume & Issue: دوره 5 - شماره 4

Title: بررسی شیوع و عوامل مؤثر در ایجاد تأخیر تکامل حرکتی در شیرخواران پرخطر

http://rehabilitationj.uswr.ac.ir/article-1-101-fa.html

هدف: شیرخواران در معرض خطر ایجاد تأخیر تکامل، شیرخواران پرخطر نامیده می‌شوند که دارای سابقه وجود یک یا چند عامل خطرساز در دوران قبل یا پس از تولد هستند. میزان شیوع تأخیر تکامل در این شیرخواران بالاتر از جمعیت عادی است. این مطالعه با هدف تعیین شیوع تأخیر تکامل حرکتی و عوامل خطرساز مؤثر در ایجاد آن در شیرخواران پرخطر مراجعه‌کننده به مرکز اختلالات تکاملی صبا انجام شده است. روش بررسی: مطالعه به‌صورت توصیفی (تعیین شیوع) و تحلیلی از نوع مورد شاهدی (تعیین عوامل خطرساز مؤثر) انجام شده است. جامعه تحقیق شامل 403 شیرخوارار ارجاع‌شده از مراکز بهداشت و درمانگاه‌های تخصصی اطفال تهران است که دارای سابقه یک یا بیشتر عوامل خطرساز برای ایجاد تأخیر تکامل بوده‌اند. موارد دچار تأخیر تکامل به‌عنوان مورد و شیرخواران مراجعه‌کننده به مرکز به‌علل دیگر، به‌عنوان شاهد تلقی گردیدند. یافته‌ها: براساس این مطالعه مشخص گردید شیوع تأخیر تکامل حرکتی در شیرخواران پرخطر %30 و به‌طور قابل توجهی بالاتر از جمعیت عادی است. همچنین مؤثرترین عوامل خطرساز به‌ترتیب نارس‌بودن هنگام تولد (%6/25) وزن کم حین تولد (%19) تشنج‌های نوزادی (%4/7) بیماری غشاء هیالن (%6/6) عفونت‌های سیستمیک مادر در دوران بارداری (%7/5) و زردی شدید نوزادی (%9/4) هستند. نتیجه‌گیری: با توجه به مؤثربودن عوامل فوق در ایجاد شیوع بالاتری از تأخیر تکامل حرکتی در شیرخواران پرخطر، در جهت پیشگیری از این عوامل و در نتیجه، کاهش شیوع تأخیر تکامل بایستی اقدام نمود. همچنین باید در جهت شناسایی هرچه سریع‌تر شیرخواران مبتلا به تأخیر تکامل به‌منظور انجام مداخلات درمانی و توان‌بخشی به‌موقع اقدام نماییم تا بدین‌ترتیب به نتایج درمانی بهتری در آنان دست خواهیم یافت.

Title: کاربردهای آزمایشگاهی آزمون‌های تشخیصی در آسیب‌شناسی گفتار

http://rehabilitationj.uswr.ac.ir/article-1-102-fa.html

هدف: در این تحقیق کاربردهای آزمایشگاهی آزمون‌ها و معیارهای ارزیابی برای سنجش مشخصه‌های آسیب‌شناسی گفتار برای تشدید حفره‌ای و نیمرخ حرکتی گفتار (MSP) در دو دستگاه آزمایشگاهی اسنورز (SNORS) و ویزی‌پیچ (VISIPITCH III) برای گویندگان فارسی‌زبان بررسی شد. روش بررسی: با توجه به اینکه آزمون‌های ارزیابی موجود برای زبان انگلیسی تدوین شده بود به منظور استفاده در ارزیابی های آزمایشگاهی در زبان فارسی ابتدا آزمون‌ها براساس مشخصات آوایی زبان فارسی تدوین و هنجارسازی ساختاری شد. سپس به‌منظور کاربردهای آزمایشگاهی و تشخیصی برای دستگاه اسنورز در 15 نفر و دستگاه ویزی‌پیچ در 31 نفر گویندگان سالم فارسی‌زبان در محدوده سنی 19 تا 37 سال هنجاریابی شد. یافته‌ها: نتایج به‌دست‌آمده در دستگاه اسنورز با جدول هنجار موجود برای هر واژه در بررسی میزان خیشومی‌شدگی مقایسه شد و با توجه به انحراف معیار کمتر از %5 مورد قبول قرار گرفت و در دستگاه ویزی‌پیچ جمله‌های پیشنهادی با P=0 و انحراف معیار STD=33/06 و تفاوت میانگین 165/38 مورد تأیید قرار گرفت. نتیجه‌گیری: براساس نتایج آماری موجود از آزمون‌های تشخیصی دو دستگاه مذکور می‌توان برای ارزیابی‌های آزمایشگاهی تشدید حفره‌ای و نیمرخ حرکتی گفتار در گویندگان فارسی‌زبان استفاده کرد.

Title: بررسی علل ضایعات نخاعی مددجویان استفاده‌کننده از خدمات توان‌بخشی کشور

http://rehabilitationj.uswr.ac.ir/article-1-103-fa.html

هدف: افزایش صدمات ناشی از حوادث زندگی ماشینی موجب افزایش روزافزون ضایعات نخاعی شده است. به‌منظور بالابردن دانش بهداشتی جامعه در جهت کاهش معلولیت و بهبود کیفیت زندگی مددجویان ضایعات نخاعی، تحقیق حاضر طراحی و در 24 استان کشور به انجام رسید. روش بررسی: در طرح میدانی حاضر 1426 مددجوی ضایعه نخاعی در سطح 24 استان مورد بررسی قرار گرفتند0پرسشنامه جامعی مشتمل بر توزیع فراوانی، علل معلولیت، استفاده از خدمات فیزیوتراپی ، توانبخشی و بهزیستی و حجم این خدمات در مورد هر مددجو تکمیل و مورد تجزیه و تحلیل قرار گرفت. یافته‌ها: 2/82 درصد از ضایعات نخاعی در سطح کشور در اثر سوانح، حوادث و جنگ رخ داده است. 4/58 درصد از مددجویان از خدمات فیزیوتراپی استفاده کرده‌اند که در 4/29 آنها این خدمات کمتر از 2 ماه بوده است. 5/61 درصد از مددجویان از خدمات سازمان بهزیستی استفاده کرده‌اند که از این بین، 2/36 درصد از خدمات رفاهی، 9/33 درصد از وسایل کمک توان‌بخشی و 4/22 درصد از خدمات بهداشتی درمانی استفاده کرده‌اند. نتیجه‌گیری: بیشتر ضایعات نخاعی در کشور در اثر حوادث اکثراً قابل پیشگیری اتفاق می‌افتد. علی‌رغم نیاز شدید مددجویان به انجام خدمات فیزیوتراپی و توان‌بخشی، تعداد قابل توجهی از آنها از دسترسی به خدمات فیزیوتراپی و توان‌بخشی محروم بوده‌اند. امید است سازمان بهزیستی کشور -که به‌عنوان متولّی امور معلولین نقش قابل توجهی در توان‌بخشی و حمایت مددجویان داشته- با حمایت مدیریت کلان کشور تقویت و سامان‌دهی شود.

Title: بررسی میزان افسردگی اساسی در کودکان و نوجوانان مبتلا به اختلال تورت

http://rehabilitationj.uswr.ac.ir/article-1-104-fa.html

هدف: اختلال تورت عبارت است از اختلالی که در آن سابقه تیک‌های حرکتی متعدد در بیمار وجود دارد و حداقل یک تیک صوتی در مقطعی از اختلال در بیمار ایجاد می‌شود. گزارشات فراوانی در مورد همراهی افسردگی اساسی به‌طور هم‌زمان با این اختلال منتشر شده و مطالعات بیانگر این است که تشخیص و درمان به‌موقع این اختلال از اهمیت بسیار زیادی در سیر درمان بیماران مبتلا به اختلال تورت برخوردار است. بنابراین، بررسی افسردگی اساسی هم‌زمان با اختلال تورت در جامعه اهمیت فراوانی دارد. روش بررسی: در طی یک دوره زمانی یکساله، 30 مورد بیمار مبتلا به اختلال تورت مراجعه‌کننده به یک مرکز روان‌پزشکی اطفال مورد بررسی قرار گرفتند. این مطالعه به‌صورت توصیفی و Cross Sectional طراحی شد. همچنین 30 نفر از افراد گروه کنترل مطابق با گروه بیماران از لحاظ سن و جنس از مدارس شهر تهران انتخاب شدند. 25 پسر و 5 دختر حدود سنی 18-8 سال در هر گروه وجود داشتند. پرسشنامه K-SADS جهت یافتن افسردگی اساسی در این بیماران و همچنین در گروه کنترل تکمیل گردید. نتایج به‌دست‌آمده در این تحقیق بر اساس آزمون‌های آماری از جمله MC-Nemar Exact Test مورد تجزیه و تحلیل قرار گرفت. یافته‌ها: %3/23 از بیماران مبتلا به اختلال تورت و %3/3 از افراد گروه کنترل به اختلال افسردگی اساسی مبتلا بودند. نتیجه‌گیری: همراهی قابل ملاحظه بین اختلال تورت و اختلال افسردگی اساسی در این تحقیق ایجاب می‌نماید که هر بیمار مبتلا به اختلال تورت به‌طور جدی از نظر افسردگی اساسی بررسی شود؛ زیرا می‌تواند روی برنامه درمان و اداره بیماری تأثیرگذار باشد.

Title: تعیین حساسیت و اعتبار افتراقی آزمون‌های تحملی عضلات تنه در افراد سالم و بیماران مبتلا به کمردرد

http://rehabilitationj.uswr.ac.ir/article-1-105-fa.html

هدف: این تحقیق برای تعیین میزان حساسیت آزمون‌های مختلفی که به‌منظور بررسی تحمل عضلات خلف کمر در افراد مبتلا به کمردرد مورد استفاده قرار می‌گیرد انجام یافت، تا از میان آنها بتوان مهم‌ترین، کلینیکی‌ترین و مقرون‌به‌صرفه‌ترین آزمون را انتــخاب کرد. این آزمون شامـل: آزمون‌های Supine Isometric Chest Raise، Supine Double SLR، Prone Isometric Chest Raise، Prone Double SLR و آزمون Sorensen هستند. بر مبنای فرضیات، یافته‌های بالینی و نتایج تحقیقات گذشته کاهش تحمل عضلات تنه، نقش بسیار مهمی در بروز کمردرد دارند. به اعتقاد محققین کاهش تحمل عضلات تنه و خستگی زودرس این عضلات موجب اعمال فشار بیش‌ازحد معمول روی عناصر پاسیو ستون فقرات کمری، به‌هم‌خوردن تعادل نیرو و ارتباط طبیعی مهره‏‌ها، کاهش توانایی مقابله با نیروهای غیرمنتظره وارد بر ستون فقرات و آسیب‌دیدگی عناصر حساس به درد می‌شود و بکمردرد را بروز می‌دهد. بنابراین، نحوه آزمون و اندازه‌گیری تحمل این عضلات در پیشگیری، درمان و توان‌بخشی این عضلات بسیار مهم و ضروری به‌نظر می‌رسد. با توجه به وجود روش‌های متعدد بالینی به‌منظور ارزیابی تحمل عضلات تنه و یافتن مهم‌ترین و حساس‌ترین آزمون از میان آنها می‏‌تواند تاحد زیادی در ارزیابی و تشخیص بیماران مبتلا به کمردرد کمک کند. روش بررسی: 200 نفر در این تحقیق شرکت داشتند و افراد به چهار گروه مساوی مردان سالم (50 نفر)، زنان سالم (50 نفر)، مردان مبتلا به کمردرد (50 نفر) و زنان مبتلا به کمردرد (50 نفر) تقسیم شدند. آزمون‌های موردنظر در هر گروه اندازه گرفته و میزان ارتباط آنها با بروز کمردرد سنجیده شد. یافته‌ها: نتایج حاصل از آزمون ICC در مورد آزمون‌های فوق نشان می‌دهد که کلیه آزمون‌ها از تکرارپذیری نسبتاً خوبی برخوردارند. آزمون Sorensen بین افراد سالم و بیمار اختلاف معنی‌داری نداشت. ‌آزمون‌های دیگر بین مردان و زنان سالم و افراد بیمار اختلاف معنی‌دار دارد. نتیجه‌گیری: نتایج حاصله از تحلیل رگرسیون لوجستیک (Backward Logistic Regression) نشان داد از میان ‌آزمون‌های مورد بررسی، آزمون Prone double SLR بیشترین ارتباط را با بروز کمردرد دارد و دیگر آزمون‌ها ارتباط معنی‏‌دار ضعیف‌تری با بروز کمردرد دارند.

Title: بررسی تأثیر مراقبت پرستاری در منزل بر عوارض ناشی از بی‌حرکتی در سیستم عضلانی اسکلتی بیماران مبتلا به سکته مغزی بستری در منزل در شهرستان سمنان 82-1381

http://rehabilitationj.uswr.ac.ir/article-1-107-fa.html

هدف: سکته مغزی عامل ایجاد عوارض طولانی‌مدت در بیماران محسوب می‌شود. با توجه‌ به عوارض ناشی از بی‌حرکتی در سکته مغزی‌، یکی‌ از مهم‌ترین‌ مسائلی‌ که لازم است‌ به‌ آن‌ توجه‌ نمود، مراقبت پرستاری از بیمار است. هدف از این مطالعه تعیین تأثیر مراقبت در منزل بر عوارض ناشی از بی‌حرکتی در سیستم عضلانی اسکلتی بیماران مبتلا به سکته مغزی بستری در منزل بوده است. روش بررسی: در این مطالعه نیمه‌تجربی، جامعه پژوهش کلیه بیماران مبتلا به سکته مغزی بستری در منزل هستند که در زمستان سال 81 و بهار 82 در یکی از بیمارستان‌های شهرستان سمنان بستری بوده‌اند که از بین آنها تعداد 32 نفر به‌صورت تصادفی انتخاب گردیده‌اند (آزمون و شاهد). ابزار گردآوری داده‌ها شامل پرسشنامه‌ای مشتمل بر دو بخش است: بخش اول مشخصات دموگرافیک و بخش دوم به صورت چک‌لیست بررسی عوارض ناشی از بی‌حرکتی. سپس برای بیماران گروه آزمون مراقبت‌های پرستاری با تأکید بر سیستم‌های عضلانی اسکلتی هر هفته 12 تا 18 جلسه (هر جلسه به‌مدت 45 دقیقه تا یک ساعت) جمعاً به مدت 40 روز انجام گرفته است. یافته‌ها: تجزیه و تحلیل اطلاعات به‌دست‌آمده با استفاده از آمار توصیفی و استنباطی و مقایسه عوارض ناشی از بی‌حرکتی بعد از انجام مداخلات بین دو گروه آزمون و شاهد در سیستم عضلانی اسکلتی (P=0/006)، اختلاف آماری معنی داری را نشان می‌دهد. همچنین در مورد فعالیت‌های روزمره زندگی، ضعف عضلانی و وضعیت فعالیت حرکتی در گروه آزمون نسبت به شاهد بهبود مشاهده شد. نتیجه‌گیری: یافته‌های حاصل در ارتباط با هدف کلی پژوهش نشان داد که میزان عوارض ناشی از بی حرکتی در گروه آزمون نسبت به گروه شاهد کمتر بوده است؛ یعنی براساس فرضیه، انجام مراقبت‌های پرستاری در منزل بر عوارض ناشی از بی‌حرکتی در سیستم‌های عضلانی اسکلتی مؤثر بوده و نقش بسزایی در کاهش عوارض ناشی از بی‌حرکتی در سیستم مذکور داشته است.

Title: تأثیر تعدیل حسی کف پا در کاهش اسپاستی‌سیته اندام تحتانی کودکان دی‌پلژی‌اسپاستیک

http://rehabilitationj.uswr.ac.ir/article-1-108-fa.html

هدف: هدف از این مطالعه، بررسی نقش تعدیل حسی کف پا در کاهش اسپاستی‌ستیه اندام تحتانی کودکان فلج مغزی دی‌پلژی‌اسپاستیک است. روش بررسی: این بررسی روی 24 کودک در دامنه سنی 5/4-5/2 سال در دو گروه کنترل و مورد صورت گرفت. ارزیابی تونوس عضلانی به‌وسیله معیار آشوورت تغییریافته (Modified Ashworth)، دامنه حرکتی غیرفعال به‌وسیله گونیامتر باساس روش پدرتی (Pedretti) و سطح مرحله رشد حرکتی توسط معیار بوبت انجام شد. تمام کودکان در طی 10 هفته، دو بار (یک‌بار قبل از درمان و یک‌بار در پایان مدت درمان) مورد ارزیابی قرار گرفتند. یافته‌ها: کاهش اسپاستی‌سیته اکستانسور ران (P<0/01) و پلانتار فلکسور مچ پا (P>0/05) در گروه مورد بیشتر از شاهد بود. افزایش دامنه حرکتی غیرفعال فلکشن ران (P>0/01)، اکستنشن زانو (P<0/05) و دورسی فلکشن مچ پا (P>0/05) در گروه مورد بیشتر از شاهد بود. اختلاف معنی‌داری در کاهش اسپاستی‌سیته فلکسور زانو و بهبود سطح مرحله رشد حرکتی وجود نداشت. درمقابل، ارتباط معنی‌داری بین کاهش اسپاستی‌سیته اکستانسور ران با افزایش دامنه حرکتی غیرفعال فلکشن ران (P>0/05) و بهبود سطح مرحله رشد حرکتی (P>0/05) به‌دست آمد. نتیجه‌گیری: استفاده از تعدیل حسی کف پا در کودکان دی‌پلژیک‌اسپاستیک باعث کاهش اسپاستی سیته در اندام تحتانی و در نتیجه افزایش دامنه حرکتی مفاصل اندام می‌گردد.

Title: بررسی تأثیر آرام‌سازی پیشرونده عضلانی بر فشارخون سالمندان مبتلا به هیپرتانسیون اولیه ساکن در مرکز سالمندان کهریزک در سال 1383

http://rehabilitationj.uswr.ac.ir/article-1-109-fa.html

هدف: هیپرتانسیون یکی از بیماری‌های مزمن است که از نظر هزینه درمانی و مراقبتی، رقم بالایی را به خود اختصاص می‌دهد و شایع‌ترین بیماری قلبی–عروقی به‌حساب می‌آید. به‌علت شیوع بالا (بیش از 60 درصد در افراد بالای 65 سال) این بیماری و افزایش خطر بیماری‌های قلبی–عروقی ناشی از آن یک مشکل عمده بهداشتی است؛ لذا با توجه به موارد مذکور و مزایای روش‌های غیردارویی مؤثر بر فشارخون، پژوهشگر انجام تحقیق حاضر را ضروری می‌داند تا تأثیر برنامه آرام‌سازی پیشرونده عضلانی را روی فشارخون سالمندان مبتلا به هیپرتانسیون اولیه مورد بررسی قرار دهد. روش بررسی: این پژوهش یک پژوهش نیمه‌تجربی است که در دو گروه آزمون و شاهد به‌منظور بررسی تأثیر تکنیک آرام‌سازی پیشرونده عضلانی بر فشارخون سالمندان مبتلا به هیپرتانسیون اولیه ساکن در مرکز سالمندان کهریزک در سال 1383 صورت گرفته است. تعداد 55 نفر سالمند هیپرتانسیونی (27 نفر گروه آزمون و 28 نفر گروه شاهد) به روش نمونه‌گیری تصادفی مورد مطالعه قرار گرفتند. ابزار گردآوری داده‌ها شامل پرسشنامه اطلاعات دموگرافیک، چک‌لیست ثبت فشارخون و تعداد ضربان نبض و دستگاه فشارسنج و گوشی پزشکی بوده است. برای گروه آزمون تکنیک آرام‌سازی به‌مدت شش هفته و هفته‌ای سه روز، در طی جلسات 20 دقیقه‌ای اجرا می‌شد و میزان فشارخون افراد در هر بار پس از اجرای مداخله، مورد بررسی قرار می‌گرفت و در پایان با گروه شاهد مقایسه می‌گردید. تجزیه و تحلیل آماری از طریق نرم افزار‌آماری SPSS صورت پذیرفت. یافته‌ها: آزمون آماری من-‌ویتنی نشان داد فشار خون سیستولیک و دیاستولیک در گروه آزمون و شاهد قبل از مداخله اختلاف معنی‌داری باهم نداشته‌اند ( P<0.05) نتیجه‌گیری: نتایج نهایی پژوهش نشان می‌دهند که فشارخون سیستول و دیاستول در گروه آزمون کاهش یافته است. با توجه به این نتایج می‌توان گفت که فرضیه پژوهش با عنوان «تکنیک آرام‌سازی باعث کاهش فشارخون در افرادی می‌شود که این تکنیک را تمرین می‌کنند» مورد تأیید قرار می‌گیرد.

Title: بررسی شیوع و علل حوادث چشمی ناشی از کار در کارگاه‌های صنعتی مشهد

http://rehabilitationj.uswr.ac.ir/article-1-110-fa.html

هدف: حوادث شغلی یکی از عوامل تخریب شدید ساختمان چشم محسوب می‌شود و می‌تواند آسیب دائمی در عمل بینایی ایجاد نماید. به‌منظور تجزیه و تحلیل حوادث چشمی ناشی از کار و ارائه طرح‌ها و پیشنهادات مؤثر جهت کاهش و پیشگیری از آن‌، 1048 پرونده حادثه شغلی مربوط به سال‌های 81-78 13 به‌طور تصادفی مورد بررسی قرار گرفتند. روش بررسی: مشخصات فردی، نحوه و چگونگی بروز حوادث چشمی 63 بیمار از فرم‌های گزارش حوادث سازمان تأمین اجتماعی مربوط به کارگاه‌های صنعتی مشهد استخراج و با نرم‌افزار اکسل و آزمون مجذور کای تجزیه و تحلیل شدند. یافته‌ها: 6/05 درصد از حوادث شغلی مربوط به چشم بود. ارتباط معنی‌داری بین سن و علت وقوع حادثه چشمی در این تحقیق پیدا نشد. علت وقوع 8/28 درصد حوادث بی‌احتیاطی و عدم استفاده از وسایل حفاظتی بین 20 تا 40 سالگی بود. 7/25 درصد از حوادث، مربوط به ورود اجسام خارجی به چشم بین 20 تا 40 سالگی بود. بین وقوع حوادث چشمی در فصول مختلف سال و نوبتِ کاری ارتباط معنی‌داری وجود داشت (0/05>P). بین نوع حادثه و فصول سال ارتباط معنی‌داری یافت نشد و 2/26 درصد ورود اجسام خارجی به چشم در نوبتِ کاری صبح اتفاق افتاد. نتیجه‌گیری: با توجه به نتایج به‌دست‌آمده به‌نظر می‌رسد که آموزش کارگران، بالابردن سطح آگاهی و مهارت‌های شغلی، استفاده صحیح از وسایل حفاظتی فردی و گروهی، اجتناب از به‌کارگیری وسایل معیوب، پرهیز از اضافه‌کاری و به‌کارگیری روشنایی مناسب برای کاهش حوادث شغلی از اولویت برخوردار باشند.

Title: گزارش یک خانواده ایرانی مبتلا به سندرم پندرد با جهش جدید T420I و نیز جهش هتروزیگوت مرکب T420I و 1197delT

http://rehabilitationj.uswr.ac.ir/article-1-111-fa.html

شیوع ناشنوایی مادرزادی در حدود 1 در هر 1000 تولد زنده است. بیش از 50 جایگاه ژنتیکی مجزا شناخته شده است که جهش در آنها منجر به ناشنوایی می‌شود. DFNB4 یک جایگاه ژنی مغلوب شناخته‌شده برای ناشنوایی است که جهش در آن باعث سندرم ناشنوایی پندرد می‌شود. ناشنوایی عصبی، پیش ‌کلامی است و در برخی موارد پیشرونده و منجر به درجات مختلفی از ناشنوایی می‌شود. در این مطالعه یک خانواده مبتلا به ناشنوایی گزارش می‌شود که در آن سه فرزند مبتلا به سندرم پندرد با جهش جدیدی به صورت هموزیگوت، مشاهده شده است. فرزندان خانواده 20، 23 و 32 ساله بوده و هر سه مبتلا به ناشنوایی مادرزادی پیشرونده و گواتر یوتیروتید بودند، ولی جهش‌های شناخته‌شده قبلی در آنها مشاهده نشد و جهش جدید T420I به‌صورت هموزیگوت موجب سندرم پندرد در آنها شده است. والدین آنها دارای رابطه خویشاوندی و برای این جهش هتروزیگوت بودند و فنوتیپ طبیعی داشتند.
